# Phylogenetic evidence of the intercontinental circulation of a Canine distemper virus lineage in the Americas

**DOI:** 10.1038/s41598-019-52345-9

**Published:** 2019-10-31

**Authors:** July Duque-Valencia, Norma R. Forero-Muñoz, Francisco J. Díaz, Elisabete Martins, Paola Barato, Julian Ruiz-Saenz

**Affiliations:** 1 Grupo de Investigación en Ciencias Animales - GRICA, Facultad de Medicina Veterinaria y Zootecnia, Universidad Cooperativa de Colombia, Bucaramanga, Colombia; 2 Corporación Patología Veterinaria (Corpavet), Bogotá, Colombia; 3 Grupo Inmunovirología, Facultad de Medicina, Universidad de Antioquia, Calle 70 No. 52-21, Medellín, Colombia; 4 Universidade de Lisboa, Lisboa, Portugal

**Keywords:** Viral epidemiology, Viral evolution

## Abstract

Canine distemper virus (CDV) is the cause of a multisystem disease in domestic dogs and wild animals, infecting more than 20 carnivore and non-carnivore families and even infecting human cell lines in *in vitro* conditions. Phylogenetic classification based on the hemagglutinin gene shows 17 lineages with a phylogeographic distribution pattern. In Medellín (Colombia), the lineage South America-3 is considered endemic. Phylogenetic studies conducted in Ecuador using fragment coding for the fusion protein signal peptide (Fsp) characterized a new strain belonging to a different lineage. For understanding the distribution of the South America-3 lineage in the north of the South American continent, we characterized CDV from three Colombian cities (Medellín, Bucaramanga, and Bogotá). Using phylogenetic analysis of the hemagglutinin gene and the Fsp region, we confirmed the circulation of CDV South America-3 in different areas of Colombia. We also described, for the first time to our knowledge, the circulation of a new lineage in Medellín that presents a group monophyletic with strains previously characterized in dogs in Ecuador and in wildlife and domestic dogs in the United States, for which we propose the name “South America/North America-4” due its intercontinental distribution. In conclusion, our results indicated that there are at least four different CDV lineages circulating in domestic dogs in South America: the Europe/South America-1 lineage circulating in Brazil, Uruguay, and Argentina; the South America-2 lineage restricted to Argentina; the South America-3 lineage, which has only been reported in Colombia; and lastly an intercontinental lineage present in Colombia, Ecuador, and the United States, referred to here as the “South America/North America-4” lineage.

## Introduction

Canine distemper virus (CDV) belongs to the *Paramyxoviridae* family, genus *Morbillivirus*, which includes viruses with epidemiological relevance to human and animal populations^1,2^. The etiological agent of a highly prevalent viral infectious disease of domestic and wild carnivores, CDV poses a conservation threat to endangered species worldwide^3,4^.

Clinical symptoms in all affected species are influenced by strain virulence, environmental conditions, host age, and immune status. The gastrointestinal tract and the respiratory and nervous systems are the most affected in all species^5,6^. Clinical signs of CDV in dogs include the onset of a cutaneous rash, serous nasal and ocular discharge, conjunctivitis, and anorexia, followed by gastrointestinal and respiratory signs, which are often complicated by secondary bacterial infections and neurological disorders^7^.

Of the six encoded viral proteins, the hemagglutinin (H) and fusion (F) glycoproteins are responsible for virus attachment to and fusion with the host cells^8^. The H protein is of principal importance as it determines viral tropism *in vivo* and *in vitro* and thus determines host-specific immunity^9^.

Due to external pressures on the host immune system, the H gene displays higher genetic variability when compared with other CDV genes^9^, which makes it suitable for lineage identification and phylogenetic analysis. Moreover, researchers have established that a short region of the F gene, which encodes the signal peptide of the F protein (Fsp), is extremely variable, suggesting that this region could also be a useful marker for evolutionary studies as it allows for the straightforward identification of CDV lineages^10–14^.

Phylogenetic studies based on the complete sequence of the H gene or the Fsp-coding region of several CDV viruses worldwide have revealed a geographical pattern of genetic diversity. According to this pattern, there are multiple distinct lineages/genotypes, most of which follow a geographical pattern of distribution. These lineages are known as America-1 (which includes the commercially available vaccines), America-2-5, Arctic-like, Asia-1-4, Africa-1 and -2, European Wildlife, Europe/South America-1, and South America-2 and -3^9,15–24^.

Although immunization with attenuated vaccines has been widely used for preventing CDV, researchers have hypothesized that the strong genetic diversity and wide variability of the H gene could mean that the antigenic profile of these new genetic variants is altered relative to the vaccine strain if specific sites on the H protein associated with immune neutralization are affected^9,25,26^. In fact, recent analysis has suggested the need for developing an updated CDV vaccine due to differences in cross-neutralization assays revealing wide antigenic differences among wild-type CDV isolates and the vaccine strains currently used in the U.S.^27^.

Different publications have shown a worldwide increase in the incidence of the disease, even in vaccinated dog populations^12,17,28,29^ and as most CDV commercial vaccines are formulated with strains belonging to the America-1 lineage^21^, antigenic differences could possibly explain the worldwide increase in the incidence of the disease even in vaccinated dogs.

Researchers have suggested that the South American continent has one of the highest CDV antigenic variabilities in the world^13^. Phylogenetic analyses based on the CDV H gene from South America have been performed for establishing the evolutionary patterns of the virus in the region and have revealed multiple circulating lineages of CDV, each differing in prevalence. In Brazil, Uruguay, and Argentina, the most prevalent lineage is Europe/South America-1^20,28^; the second-most prevalent, known as South America-2, is restricted to Argentinian canine populations and appears to be associated with strains isolated from wild carnivore species in Europe^30^.

In the northern part of South America, the circulation of different lineages has been reported; in Colombia, a third lineage (South America-3) that causes disease even in vaccinated dogs was identified on the basis of complete H gene sequencing^17^. In the same year, a possible different CDV lineage was described based on analysis of the Fsp-coding region of Ecuadorian strains^11^. Owing to differences in methodologies, complete H gene sequences are not comparable with Fsp-coding region sequences available from this region of the continent. It is possible that a genetic relationship exists between CDV strains from Colombia and Ecuador because they have similar geographic distributions in the northern region of South America.

For determining the genetic diversity of CDV from Colombia in relation to CDV from other regions of South America, we analyzed the genetic diversity within the Fsp-coding region and H gene of CDV strains currently circulating in different regions of Colombia and compared it with that of wild-type CDV circulating throughout America and worldwide in addition to vaccine strains.

## Materials and Methods

### Ethical considerations

This study was approved by the Ethics Committee for Animal Experimentation of the Universidad Cooperativa de Colombia in Bucaramanga. All experiments were performed in accordance with relevant guidelines and regulations. Dog owners signed informed consent forms approved by the ethics committee. Viral samples are subject of contract for access to genetic resources and derived products No. 132 of 2016 RGE0177 signed with the Ministry of Environment and Sustainable Development of the Republic of Colombia. In addition, the authors declare that the implementation of this work followed all scientific, technical, and administrative rules for animal research.

### Clinical specimens and vaccine strains

A total of 86 clinical samples from dogs exhibiting signs suggestive of CDV were obtained from three main Colombian provinces (Supplemental Material Fig. S1). Of these, 48 clinical specimens were taken from Bogotá D.C. between January 2014 and June 2015, 11 were taken from Bucaramanga between June 2016 and November 2016, and 27 were taken from Medellín between May and September 2017. Samples were taken in different veterinary hospitals and basic data were gathered at the time of sampling, including the dogs’ age, sex, breed, vaccination status, municipality and/or neighborhood of origin, and clinical signs. Clinical specimens included serum and ocular discharge.

### RNA extraction

Total RNA was extracted from 140 µl of the supernatant of ocular discharge, serum, and four commercial CDV vaccines using QIAamp Viral RNA Mini Kit (QIAGEN®, Hilden, Germany) in accordance with the manufacturer’s instructions. The quality and quantity of the RNA was determined using spectrophotometric analysis with a NanoDrop™ One UV-Vis Spectrophotometer (Thermo Scientific, Wilmington, Delaware, USA), and RNA aliquots were stored at −80 °C until use.

### Complementary cDNA synthesis

Complementary DNA (cDNA) was synthesized using RevertAid™ Premium First Strand cDNA Synthesis Kit (Thermo Scientific®, Glen Burnie, MD) in accordance with the manufacturer´s instructions. A denaturation mix consisting of 1 µl (100 pmol/µl) random hexamers, 1 µl dNTP Mix (10 mM) and 13 µl (0.02–4.6 µg) total RNA was initially denatured at 65 °C for 5 min and immediately incubated on ice. The RT mix solution consisted of 4 µl 5X Reverse Transcriptase Buffer and 1 µl RevertAid™ Premium Enzyme Mix. The RT mix was added to the denaturation mix and reverse transcription was performed in a total volume of 20 µl in a ProFlex™ PCR Thermal Cycler (Applied Biosystems®, Foster city, California, USA) for 10 min at 25 °C followed by 30 min at 50 °C; the reaction was terminated by heating to 85 °C for 5 min. The reaction product was stored at −80 °C until use. Commercially available vaccines were used as positive controls for RT-PCR reactions.

### PCR and sequencing

Next, cDNAs from clinical specimens were screened by PCR of the phosphoprotein (P) gene using the Maxima Hot Start PCR Master Mix (2X) (Thermo Scientific®) reagent kit in accordance with the manufacturer’s instructions. Viral cDNA was detected using morbillivirus universal primers^31^ for amplifying a 429 bp fragment of the phosphoprotein gene. For PCR, 4 µl cDNA was added to a reaction mix, which consisted of 25 µl Maxima Hot Start PCR Master Mix (2X), 15 µl nuclease-free water, and 3 µl (10 µM) of each of the forward and reverse primers. PCR was performed on a ProFlex™ PCR Thermal Cycler (Applied Biosystems®) under the following conditions: initial denaturation at 95 °C for 4 min followed by 35 cycles of denaturation at 95 °C for 30 s, annealing at 50.8 °C for 30 s, extension at 72 °C for 1 min, and a final extension at 72 °C for 5 min. Ultrapure water was used as a negative control and cDNA from one of the vaccines as positive control.

In all samples that tested positive for the P gene, the full-length H gene and the Fsp-coding region were amplified using Maxima Hot Start PCR Master Mix Kit in accordance with the manufacturer’s instructions. The H gene was detected using the primers CDVff1 and HS2^32^ for amplifying a 2099 bp fragment of the CDV genome that includes the H gene and flanking regions at both ends. The Fsp-coding region was amplified using the primers CDV-F4854 and CDV-R5535^10^ or F5/R5^21^ flanking the Fsp-coding region. In all cases, 4 µl cDNA was added to a PCR reaction mix, which consisted of 25 µl Maxima Hot Start PCR Master Mix (2X), 15 µl nuclease-free water and 3 µl (10 µM) of each of the primers (Table 1). PCR was performed on ProFlex™ PCR Thermal Cycler (Applied Biosystems®) under the following conditions: initial denaturation at 95 °C for 4 min followed by 35 cycles of denaturation at 95 °C for 30 s, annealing for 30 s, extension at 72 °C for 2 min, and a final extension at 72 °C for 10 min. The annealing temperature for H gen was 48.2 °C, and for the Fsp-coding region the temperature was 50.8 °C for the F5/R5 primers and 58 °C for the CDV-F4854/R5535 primers.

**Table 1:** Oligonucleotides used for CDV P gene detection and for full length H gene and Fsp-coding region amplification and sequencing.

Oligonucleotide label	Oligonucleotide sequence	Genomic position*	Reference
	**Amplification of P gene**		
CDV Universal (forward)	ATGTTTATGATCACAGCGGT	2132–2151	Daly *et al*., 2006
CDV Universal (reverse)	ATTGGGTTGCACCACTTGTC	2541–2560	Daly *et al*., 2006
	**Amplification and sequencing of H gene**		
CDVff1 (forward)	TCGAAATCCTATGTGAGATCACT	6897–6919	Lan *et al*.^32^
CDVHS2 (reverse)	ATGCTGGAGATGGTTTAATTCAATCG	8994–8969	Lan *et al*.^32^
CDVHS1 (forward)	AACTTAGGGCTCAGGTAGTCC	7054–7074	Lan *et al*.^32^
CDVHforD (forward)	GACACTGGCTTCCTTGTGTGTAG	7948–7970	Lan *et al*.^32^
CDVHr2 (reverse)	GTTCTTCTTGTTTCTCAGAGG	8198–8178	Lan *et al*.^32^
CDVP2F (forward)	ACTTCCGCGATCTCCACT	7372–7389	Pardo *et al*.^33^
CDVP3R (reverse)	ACACTCCGTCTGAGATAGC	7760–7742	Pardo *et al*.^33^
CDVP5R (reverse)	GTGAACTGGTCTCCTCTA	8395–8378	Pardo *et al*.^33^
	**Amplification and sequencing of Fsp-coding region**		
F5 (forward)	TGTTACCCGCTCATGGAGAT	4272–4292	Riley and Wilkes, 2015
R5 (reverse)	CCAAGTACTGGTGACTGGGTCT	5411–5433	Riley and Wilkes, 2015
CDV-F4854 (Forward)	TCCAGGACATAGCAAGCCAACA	4854–4875	Sarute *et al*., 2013
CDV-R5535 (Reverse)	GGTTGATTGGTTCGAGGACTGAA	5513–5535	Sarute *et al*., 2013

*Reference genome AF164967 (A75/17).

Following PCR, 5 µl amplicons were analyzed by gel electrophoresis on a 1.5% agarose gel (AGAROSE I™, Amresco, Solon, OH, USA) at 110 V for 60 min. The gels were stained using EZ-VISION™ dye (Amresco1 Solon, OH, USA) and viewed by transillumination with UV light using the Molecular Imager® GelDoc TM XR + System with the image acquisition software ImageLab™ (Bio-Rad, Hercules, CA, USA). Amplification product sizes were estimated using a 100–3000 bp molecular weight ladder (GeneRuler™ 100 bp Plus DNA Ladder, Thermo Scientific®).

PCR amplicons of the H gene and Fsp-coding region were submitted to Macrogen Inc. (Seoul, Korea) for purification and sequencing. An additional set of eight primers, published elsewhere, for the H gene were used for sequencing^32,33^ (Table 1) using ABI3711™ automatic sequencer (Macrogen™).

### Phylogenetic analysis

Sequence data were assembled and edited using SeqMan program (DNAStar Lasergene™ V15.0 software package, Madison, Wisconsin, USA). Nucleotide BLAST (Basic Local Alignment Search Tool) was used for exploring similarity between Colombian CDV strain sequences and all CDV sequences available in the NCBI nucleotide databases. For the H gene, a total length of 1824 nucleotides and corresponding deduced amino acid sequences were obtained only from dogs from Medellín and Bucaramanga, and for the Fsp-coding region (405 nucleotides and the corresponding deduced amino acid sequence), samples were obtained from all three studied cities (Bogotá DC, Medellín, and Bucaramanga). Phylogenetic analyses were carried out with at least two sequences for each reported lineage and vaccine strains from different geographical regions using MEGA™ 7^34^ and the MUSCLE algorithm, and nucleotide and amino acid differences were assessed as uncorrected (p) distances.

Phylogenetic relationships based on the nucleotide alignment of complete H gene sequences were inferred using distance-based (neighbor-joining) and character-based (maximum likelihood, Bayesian) approaches implemented in MEGA™ 7 and MrBayes 3.2.6^35^ software, respectively. The best-fit model for nucleotide substitution was identified by MEGA™ 7 as Tamura 3-parameter with gamma-distributed rate heterogeneity (T92 + G) according to the Bayesian information criterion for the H gene and Hasegawa-Kishino-Yano with gamma distribution (G) (HKY + G) for the Fsp-coding region. Maximum likelihood analysis was performed using the latter model; however, Bayesian inference analysis was performed with a general time-reversible model plus gamma-distributed rate heterogeneity (GTR + G) because the T92 + G model cannot be implemented in MrBayes 3.2.6. For this method, two parallel analyses were run for 1,000,000 generations with a 25% burn-in period. The convergence of the Markov chain Monte Carlo (MCMC) chains was assessed by the standard deviation of split frequencies, which fall below 0.01. The America-1 lineage was used as an outgroup to root the phylogenetic trees. The consensus trees were edited in FigTree software version 1.4^36^.

### Amino acid analysis of the H protein and the Fsp peptide

The deduced amino acid sequences of the H protein (607 aa) and the Fsp peptide (135 aa) of the Colombian wild-type CDV strains were aligned with multiple CDV protein sequences from different geographical regions using MEGA™ 7 for exploring their amino acid profiles and potential differences between vaccine and wild-type strains of known CDV lineages. Potential N-linked glycosylation sites were predicted using NetNGlyc 1.0^37^.

### Sites under positive selection

To identify amino acid sites under positive selection in the CDV H protein and Fsp peptide, the ratio of non-synonymous (dN) to synonymous (dS) substitutions was calculated by ML phylogenetic reconstruction using the general reversible nucleotide substitution model available through the Datamonkey web server. To detect non-neutral selection, Fast Unconstrained Bayesian AppRoximation (FUBAR) within the HyPhy software package was implemented in Datamonkey^38^. The range of significance for the posterior probability was 0–1. Generally, posterior probabilities > 0.9 are strongly suggestive of positive selection. Finally, a Bayes factor = 50 was used for estimating the rates of dN and dS within each codon. Values of dN/dS > 1, dN/dS = 1, and dN/dS < 1 were used for defining positive selection (adaptive molecular evolution), neutral mutations, and negative selection (purifying selection), respectively.

### H gene and Fsp phylogeography

The mean substitution rate (substitutions per site per year), the time to the most recent common ancestor (TMRCA), the geographic origin, and the overall spatial dynamics of the major CDV clades were inferred using the Bayesian approach of the MCMC implemented in the BEAUti/BEAST v1.8.4 package^39^. The analysis was implemented using a strict molecular clock with a constant population size, and 3E07 generations were run in order to ensure an effective population size greater than 200 for the evaluated parameters using the Tracer v1.7 program^39^. The initial 10% of the MCMC, which corresponds with low probability states at the beginning of the chain, was eliminated. The tree of maximum credibility of the MCC clades was built with TreeAnnotator and visualized with FigTree v1.4.3^40^.

## Results

### Detection of P gene and clinical features

A fragment of 429 bp of the phosphoprotein gene was detected in 68 (79.1%) clinical specimens from the 86 dogs sampled. In total, 44.1% of the CDV positive animals were male and 55.9% were female. Young dogs from one to six months old were the most affected (46.9%), although the disease also presented in dogs older than 12 months (21.9%). Concerning clinical manifestations in affected dogs, nervous and respiratory symptoms accounted for 25% of the cases, closely followed by the presentation of respiratory signs alone (21.4%). An equal proportion (17.9%) of clinically ill dogs presented with tegumentary/respiratory/nervous signs or respiratory/digestive symptoms alone.

### Sequence analysis of the H gene and the Fsp-coding region

We only were able to amplify and sequence a fragment of 2099 bp of the H gene of six clinical specimens. Next, 405 bp of the Fsp-coding region was assessed, and we were able to obtain positive amplifications and sequence 23 clinical samples out of the 68 P gene positives Samples. Information regarding the age, gender, breed, vaccination status, and clinical signs of the dogs, as well as outcome and accession numbers from H gene–positive samples, is summarized in Table 2.

**Table 2:** Clinical features of Colombian dogs infected with canine distemper virus (CDV).

	Sample^a^	Sex^b^	Age^c^	Clinical signs^d^	Vaccination status	Outcome	Gene	Region	Code GenBank
MDE 2a/CO/2017	NS	M	2Y	R, GI	Unknown	Euthanized	H,F	MEDELLIN	MK617353/MK599333
MDE 2aP/CO/2017	CS	F	6M	R,N	Unknown	Euthanized	H,F	MEDELLIN	MK617350/MK599334
MDE 1aM/CO/2017	CS	M	8Y	R	Incomplete vacination	Euthanized	H,F	MEDELLIN	MK617348/MK599332
MDE 13b/CO/2017	CS	M	7Y	R,N	Not vaccinated	Euthanized	H,F	MEDELLIN	MK617351/MK599328
MDE 16a/CO/2017	CS	F	9M	N	Not vaccinated	Euthanized	H,F	MEDELLIN	MK617352/MK599329
MDE 18 a/CO/2017	CS	M	7M	R,N	Incomplete vacination	Euthanized	H	MEDELLIN	MK617349
BUCA 12/CO/2016	CS	F	3M	R,N	Incomplete	Died	F	BUCARAMANGA	MK599327
MDE 19a/CO/2017	CS	F	5M	R	Incomplete	Unknown	F	MEDELLIN	MK599331
MDE 9a/CO/2017	CS	F	7Y	R,N	Not vaccinated	Euthanized	F	MEDELLIN	MK599337
MDE 18/CO/2012	CS	M	Unknown	R,N	Unknown	Unknown	F	MEDELLIN	MK599330
MDE 42e/CO/2012	CS	M	3M	GI,N	Not vaccinated	Unknown	F	MEDELLIN	MK599335
MDE 44/CO/2012	CS	M	5M	R,N	Unknown	Unknown	F	MEDELLIN	MK599336
CM-14–027	BLOOD	Unknown	1M	R,GI,N	Unknown	Unknown	F	BOGOTÁ	MK617339
CM-14-160	URINE	Unknown	2M	R,GI,N			F	BOGOTÁ	MK617343
CM-15-001	URINE	Unknown	Unknown	R	Unknown	Unknown	F	BOGOTÁ	MK617344
CM-15-061	URINE	Unknown	8M	R	Unknown	Unknown	F*	BOGOTÁ	—
CM-15-089	BLOOD	Unknown	4M	NS	Unknown	Unknown	F	BOGOTÁ	MK617340
CM-15-079	BLOOD	Unknown	4M	NS	Unknown	Unknown	F	BOGOTÁ	MK617341
CM-15-018	URINE	Unknown	3M	Unknown	Unknown	Unknown	F	BOGOTÁ	MK617345
CM-15-052	URINE	Unknown	14M	Unknown	Unknown	Unknown	F*	BOGOTÁ	—
CM-15-061	URINE	Unknown	8M	R	Unknown	Unknown	F*	BOGOTÁ	—
CM-15-066	URINE	Unknown	6M	R,GI,N	Unknown	Unknown	F	BOGOTÁ	MK617346
CM-15-069	BLOOD	Unknown	5M	NS	Unknown	Unknown	F	BOGOTÁ	MK617347
CM-15-078	BLOOD	Unknown	4M	NS	Unknown	Unknown	F*	BOGOTÁ	—
CM-15-135	NS	Unknown	4Y	R	Unknown	Unknown	F*	BOGOTÁ	—
CM-15-171	BLOOD	Unknown	3M	GI,T	Unknown	Unknown	F	BOGOTÁ	MK617342

a S: Serum; CS: Conjunctival swab – b F: female; M: male – c M: months; Y: Years – d R: Respiratory; GI: Gastrointestinal; O: Ocular; N: Neurological; T: Tegumentary; NS: Without Signs * Sequences don´t include in the phylogenetic analysis.

The Fsp-coding region sequences of the strains from Medellín, D/Mde_19a/CO/2017, D/Mde_2aP/CO/2017, D/Mde_13b/CO/2017, and D/Mde_16a/CO/2017, were 100% identical. Likewise, the sequences from Bogotá, D/Bog-4/CO/2015, D/Bog-5/CO/2015, and D/Bog-6/CO/2015, were 100% identical to each other. Consequently, only one of those sequences (D/Bog-4/CO/2015) were included with the rest of the Colombian strains in the phylogenetic analysis. Vaccines used as positive controls in the PCR reactions were also sequenced.

As we previously reported^17^, BLAST analysis of H sequence data from commercial Colombian vaccines used as positive controls in the present study revealed that vaccines had 99–100% identity with the vaccine strains from the North America-1 lineage, and one vaccine showed 99% identity with a Rockborn vaccine strain.

Colombian H sequences subjected to analysis displayed high identity with each other (93.5–99.9% nt; 93–99.9% aa) with an overall mean distance of 0.039. Alignment of the H gene of Colombian CDV strains and the Onderstepoort vaccine strain (AF378705) showed an identity that varied between 89.6% and 91.1% at the aa level and between 90.8% and 91.8% at the nt level. As expected, higher variability was found in the Fsp-coding region (81.39–99.01% nt, 63.91–97.7% aa) with an overall mean distance of 0.1014. Moreover, the Colombian Fsp-coding region sequences displayed very low identity with those of the Onderstepoort vaccine strain (80.9–83.6% nt, 57.6–67.4% aa).

Phylogenetic relationships based on the nucleotide alignment of complete H gene sequences inferred by distance (neighbor-joining) and character approaches (maximum likelihood and Bayesian inference) resulted in trees with similar topology. The phylogenetic tree of the H gene showed 16 lineages with a defined geographical distribution pattern (the Asia-3 lineage was grouped with strains of the America-1 lineage), while the Fsp tree only showed 15 lineages, primarily because there are no available Fsp sequences for the European Wildlife lineage.

**Figure 1:**
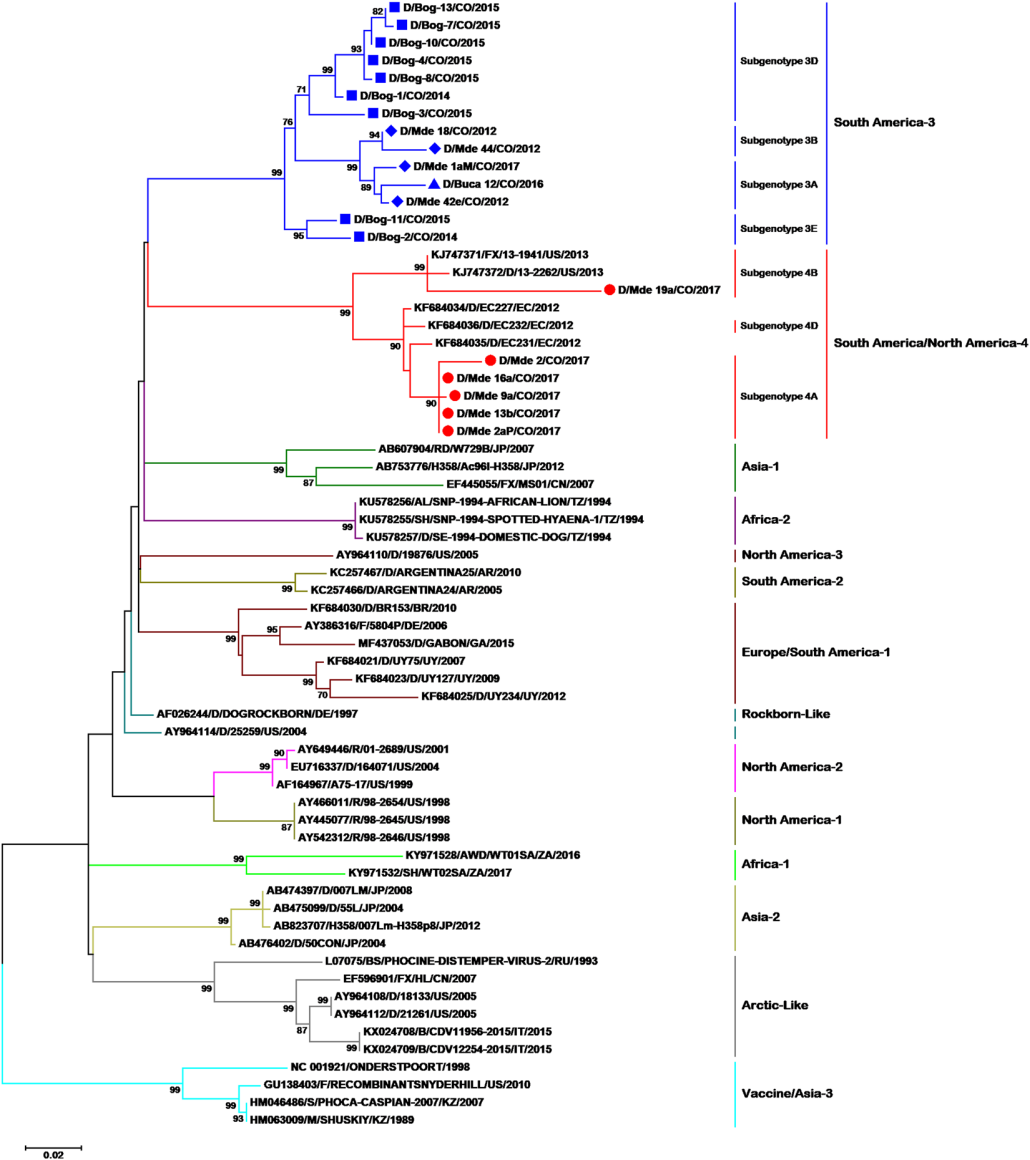
Phylogenetic relationships between 64 CDV strains based on Fsp fragment gene sequences. The phylogenetic tree was inferred by the maximum likelihood method using 1000 replicates. GenBank accession numbers, the species from which each isolate was obtained, name of the strain, country of origin, and year of isolation are indicated in the tip labels if available. Numbers at the nodes are bootstrap values for the clade. Abbreviations for animal species: AL: African lion (*Panthera leo*), AWD: African wild dog (*Lycaon pictus*), B: badger (*Meles meles*), BS: Baikal seal (*Pusa sibirica*), D: dog (*Canis lupus familiaris*), F: ferret (*Mustela putorius furo*), FX: fox (*Vulpes urocyon*), H358: human lung cells, M: mink (*Neovison vison*), R: raccoon (*Procyon lotor*), RD: raccoon dog (*Nyctereutes procyonoides*), S: seal (*Phoca vitulina*), SH: spotted hyena (*Crocuta crocuta*). Abbreviations for countries: AR: Argentina, BR: Brazil, CN: China, CO: Colombia, DE: Germany, EC: Ecuador, GA: Gabon, IT: Italy, JP: Japan, KZ: Kazakhstan, RU: Russia, TZ: Tanzania, US: United States, UY: Uruguay, ZA: South Africa.

**Figure 2:**
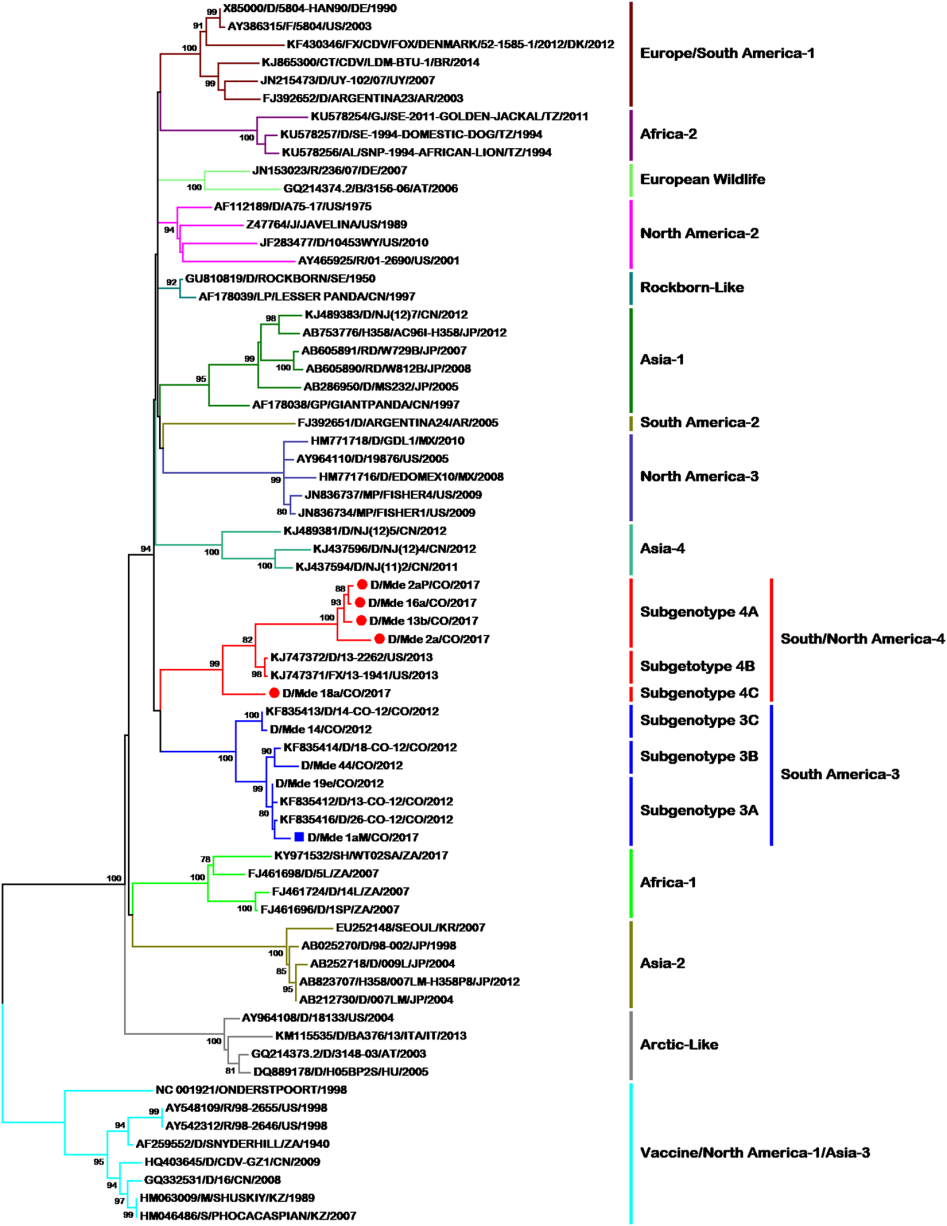
Phylogenetic relationships between 68 CDV strains based on H gene sequences. The phylogenetic tree was inferred by the maximum likelihood method using 1000 replicates. GenBank accession numbers, the species from which each isolate was obtained, name of the strain, country of origin, and year of isolation are indicated in the tip labels if available. Numbers at the nodes are bootstrap values for the clade. Abbreviations for animal species: AL: African lion (*Panthera leo*), B: badger (*Meles meles*), CT: *Cerdocyon thous*, D: dog (*Canis lupus familiaris*), F: ferret (*Mustela putorius furo*), FX: fox (*Vulpes urocyon*), GJ: golden jackal (*Canis aureus*), GP: giant panda (*Ailuropoda melanoleuca*), J: javelina (*Tayassu pecari*), LP: lesser panda (*Ailurus fulgens*), M: mink (*Neovison vison*), MP: Martes pennanti, R: raccoon (*Procyon lotor*), RD: raccoon dog (*Nyctereutes procyonoides*), S: seal (*Phoca vitulina*), SH: spotted hyena (*Crocuta crocuta*). H358: human lung cells. Abbreviations for countries: AR: Argentina, AT: Austria, BR: Brazil, CN: China, CO: Colombia, DE: Germany, DK: Denmark, HU: Hungary, IT: Italy, JP: Japan, KR: South Korea, KZ: Kazakhstan, MX: Mexico, SE: Sweden, TZ: Tanzania, US: United States, UY: Uruguay, ZA: South Africa.

Interestingly, we showed that Colombian CDV sequences cluster in two different branches in both the Fsp and H gene trees (Figs 1 and 2); one group of Colombian Fsp-coding sequences cluster in the same clade as Ecuadorian strains (Fig. 1), and also, interestingly, with two recently reported North America-4 lineage sequences^21^ (97.1% identity). Other Colombian CDV sequences cluster with the South America-3 lineage previously reported in Colombia (Fig. 2). Unfortunately, no Ecuadorian H sequences have been reported to date.

Keeping in mind that Colombian viruses showed high variability at both the nucleotide and amino acid levels, we analyzed the identity of Colombian CDV H sequences and the previously reported CDV lineages (Table 3). By analysis of uncorrected (p) distances, we found that the amino acid sequences of Colombian and Ecuadorian CDV strains of the South America-4 lineage differed by less than 4% to those of strains of the previously reported North America-4 lineage. According to this, we propose that this lineage should be termed “South America/North America-4” due its intercontinental distribution. Remarkably, we observed high variation (approximately 10%) between Colombian CDV lineages and the North America-1 lineage, which includes most of the commercial vaccine strains (Table 3).

**Table 3:** Uncorrected distances (p) observed in pairs of amino acid sequences of the Fsp and H genes between CDV lineages.

	NA-4	SA-4	SA-3	VAC	SA-2	NA-1	NA-2	NA-3	EU/SA-1	ARC-L	EU-WL	AFR-1	AFR-2	ASIA-1	ASIA-2	ASIA-3	RCK-L	ASIA-4
**NA-4**		***0***,***024***	***0***,***035***	***0***,***039***	***0***,***036***	***0***,***038***	***0***,***037***	***0***,***036***	***0***,***032***	***0***,***38***	***NA***	***0***,***038***	***0***,***037***	***0***,***035***	***0***,***038***	***0***,***041***	***0***,***033***	***NA***
		*0*,*01*	*0*,*018*	*0*,*022*	*0*,*017*	*0*,*021*	*0*,*013*	*0*,*016*	*0*,*015*	*0*,*02*	*0*,*017*	*0*,*017*	*0*,*017*	*0*,*016*	*0*,*02*	*0*,*022*	*0*,*013*	*0*,*017*
**SA-4**	**0**,**124**		***0***,***034***	***0***,***038***	***0***,***034***	***0***,***037***	***0***,***035***	***0***,***036***	***0***,***032***	***0***,***036***	***NA***	***0***,***036***	***0***,***036***	***0***,***034***	***0***,***036***	***0***,***039***	***0***,***031***	***NA***
	0,028		*0*,*018*	*0*,*022*	*0*,*017*	*0*,*021*	*0*,*014*	*0*,*016*	*0*,*016*	*0*,*02*	*0*,*018*	*0*,*017*	*0*,*017*	*0*,*016*	*0*,*02*	*0*,*022*	*0*,*014*	*0*,*017*
**SA-3**	**0**,**292**	**0**,**314**		***0***,***036***	***0***,***033***	***0***,***033***	***0***,***03***	***0***,***033***	***0***,***028***	***0***,***035***	***NA***	***0***,***035***	***0***,***033***	***0***,***031***	***0***,***034***	***0***,***038***	***0***,***028***	***NA***
	0,064	0,078		*0*,*024*	*0*,*019*	*0*,*023*	*0*,*016*	*0*,*016*	*0*,*016*	*0*,*021*	*0*,*019*	*0*,*019*	*0*,*019*	*0*,*017*	*0*,*021*	*0*,*024*	*0*,*016*	*0*,*018*
**VAC**	**0**,**371**	**0**,**405**	**0**,**367**		***0***,***037***	***0***,***033***	***0***,***037***	***0***,***038***	***0***,***035***	***0***,***037***	***NA***	***0***,***036***	***0***,***038***	***0***,***037***	***0***,***038***	***0***,***017***	***0***,***035***	***NA***
	0,111	0,12	0,132		*0*,*02*	*0*,*01*	*0*,*02*	*0*,*020*	*0*,*019*	*0*,*022*	*0*,*022*	*0*,*019*	*0*,*021*	*0*,*021*	*0*,*022*	*0*,*012*	*0*,*02*	*0*,*022*
**SA-2**	**0**,**257**	**0**,**262**	**0**,**249**	**0**,**332**		***0***,***035***	***0***,***033***	***0***,***035***	***0***,***029***	***0***,***036***	***NA***	***0***,***035***	***0***,***035***	***0***,***3***	***0***,***036***	***0***,***038***	***0***,***028***	***NA***
	0,054	0,06	0,076	0,099		*0*,*019*	*0*,*014*	*0*,*016*	*0*,*013*	*0*,*019*	*0*,*018*	*0*,*017*	*0*,*018*	*0*,*016*	*0*,*019*	*0*,*021*	*0*,*014*	*0*,*016*
**NA-1**	**0**,**284**	**0**,**324**	**0**,**255**	**0**,**228**	**0**,**239**		***0***,***026***	***0***,***036***	***0***,***032***	***0***,***035***	***NA***	***0***,***034***	***0***,***035***	***0***,***034***	***0***,***037***	***0***,***038***	***0***,***03***	***NA***
	0,105	0,112	0,127	0,04	0,09		*0*,*019*	*0*,*020*	*0*,*018*	*0*,*021*	*0*,*021*	*0*,*019*	*0*,*021*	*0*,*021*	*0*,*022*	*0*,*009*	*0*,*019*	*0*,*022*
**NA-2**	**0**,**246**	**0**,**268**	**0**,**206**	**0**,**321**	**0**,**194**	**0**,**104**		***0***,***033***	***0***,***028***	***0***,***035***	***NA***	***0***,***034***	***0***,***032***	***0***,***03***	***0***,***035***	***0***,***04***	***0***,***025***	***NA***
	0,045	0,058	0,069	0,108	0,051	0,01		*0*,*012*	*0*,*01*	*0*,*017*	*0*,*013*	*0*,*014*	*0*,*014*	*0*,*012*	*0*,*017*	*0*,*02*	*0*,*008*	*0*,*013*
**NA-3**	**0**,**261**	**0**,**285**	**0**,**251**	**0**,**313**	**0**,**228**	**0**,**231**	**0**,**201**		***0***,***03***	***0***,***037***	***NA***	***0***,***035***	***0***,***035***	***0***,***034***	***0***,***039***	***0***,***041***	***0***,***03***	***NA***
	0,043	0,059	0,056	0,1	0,049	0,094	0,044		*0*,*01*	*0*,*018*	*0*,*016*	*0*,*016*	*0*,*016*	*0*,*015*	*0*,*019*	*0*,*021*	*0*,*012*	*0*,*016*
**EU/SA-1**	**0**,**242**	**0**,**263**	**0**,**235**	**0**,**337**	**0**,**205**	**0**,**242**	**0**,**176**	**0**,**216**		***0***,***034***	***NA***	***0***,***032***	***0***,***03***	***0***,***028***	***0***,***031***	***0***,***036***	***0***,***022***	***NA***
	0,048	0,063	0,059	0,093	0,042	0,088	0,046	0,033		*0*,*017*	*0*,*015*	*0*,*014*	*0*,*015*	*0*,*014*	*0*,*017*	*0*,*019*	*0*,*01*	*0*,*013*
**ARC-L**	**0**,**355**	**0**,**344**	**0**,**318**	**0**,**323**	**0**,**296**	**0**,**261**	**0**,**259**	**0**,**294**	**0**,**287**		***NA***	***0***,***034***	***0***,***035***	***0***,***036***	***0***,***033***	***0***,***038***	***0***,***032***	***NA***
	0,085	0,099	0,106	0,121	0,081	0,114	0,083	0,078	0,078		*0*,*02*	*0*,*018*	*0*,*021*	*0*,*018*	*0*,*017*	*0*,*022*	*0*,*018*	*0*,*019*
**EU-WL**	**NA**	**NA**	**NA**	**NA**	**NA**	**NA**	**NA**	**NA**	**NA**	**NA**		***NA***	***NA***	***NA***	***NA***	***NA***	***NA***	***NA***
	0,063	0,076	0,085	0,118	0,066	0,111	0,058	0,06	0,062	0,094		*0*,*017*	*0*,*017*	*0*,*016*	*0*,*02*	*0*,*022*	*0*,*014*	*0*,*017*
**AFR-1**	**0**,**343**	**0**,**351**	**0**,**3**	**0**,**325**	**0**,**267**	**0**,**25**	**0**,**254**	**0**,**291**	**0**,**273**	**0**,**288**	**NA**		***0***,***036***	***0***,***032***	***0***,***035***	***0***,***038***	***0***,***031***	***NA***
	0,064	0,08	0,084	0,099	0,07	0,093	0,062	0,062	0,06	0,074	0,071		*0*,*018*	*0*,*016*	*0*,*018*	*0*,*02*	*0*,*014*	*0*,*017*
**AFR-2**	**0**,**275**	**0**,**291**	**0**,**237**	**0**,**312**	**0**,**252**	**0**,**241**	**0**,**189**	**0**,**241**	**0**,**202**	**0**,**252**	**NA**	**0**,**316**		***0***,***033***	***0***,***037***	***0***,***04***	***0***,***029***	***NA***
	0,064	0,079	0,083	0,109	0,056	0,107	0,06	0,061	0,064	0,1	0,073	0,078		*0*,*017*	*0*,*021*	*0*,*022*	*0*,*014*	*0*,*018*
**ASIA-1**	**0**,**274**	**0**,**302**	**0**,**245**	**0**,**343**	**0**,**215**	**0**,**256**	**0**,**204**	**0**,**254**	**0**,**223**	**0**,**322**	**NA**	**0**,**305**	**0**,**242**		***0***,***034***	***0***,***039***	***0***,***027***	***NA***
	0,05	0,063	0,068	0,109	0,079	0,106	0,048	0,048	0,049	0,085	0,064	0,064	0,066		*0*,*016*	*0*,*022*	*0*,*012*	*0*,*014*
**ASIA-2**	**0**,**308**	**0**,**307**	**0**,**27**	**0**,**317**	**0**,**248**	**0**,**226**	**0**,**203**	**0**,**271**	**0**,**221**	**0**,**251**	**NA**	**0**,**27**	**0**,**258**	**0**,**273**		***0***,***04***	***0***,***031***	***NA***
	0,079	0,093	0,099	0,112	0,09	0,112	0,078	0,077	0,073	0,069	0,088	0,069	0,089	0,056		*0*,*023*	*0*,*018*	*0*,*019*
**ASIA-3**	**0**,**384**	**0**,**408**	**0**,**388**	**0**,**071**	**0**,**328**	**0**,**254**	**0**,**343**	**0**,**321**	**0**,**336**	**0**,**352**	**NA**	**0**,**328**	**0**,**323**	**0**,**353**	**0**,**321**		***0***,***037***	***NA***
	0,102	0,113	0,124	0,045	0,054	0,025	0,099	0,091	0,084	0,112	0,108	0,09	0,106	0,103	0,109		*0*,*02*	*0*,*022*
**RCK-L**	**0**,**194**	**0**,**205**	**0**,**175**	**0**,**246**	**0**,**138**	**0**,**134**	**0**,**104**	**0**,**149**	**0**,**116**	**0**,**223**	**NA**	**0**,**205**	**0**,**152**	**0**,**164**	**0**,**159**	**0**,**246**		***NA***
	0,03	0,046	0,052	0,093	0,036	0,087	0,028	0,028	0,03	0,067	0,045	0,046	0,046	0,032	0,061	0,084		*0*,*013*
**ASIA-4**	**NA**	**NA**	**NA**	**NA**	**NA**	**NA**	**NA**	**NA**	**NA**	**NA**	**NA**	**NA**	**NA**	**NA**	**NA**	**NA**	**NA**	
	0,056	0,067	0,069	0,108	0,054	0,106	0,054	0,05	0,046	0,093	0,071	0,072	0,072	0,048	0,081	0,102	0,131	

NA-4: North America-4; SA-4: South America-4; SA-3: South America-3;VAC: Vaccine; SA-2: South America-2; NA-1:North America-1, NA-2:North America-2;NA-3: North America-3; EU/SA-1:Europe/South America-1; ARC-L:Arctic-Like; EU/WL: European Wildlife; AFR-1: Africa-1; AFR-2: Africa-2;RCK-L: Rockborn-Like. Fsp values are bold. Standard error estimates are shown (in italics) above the diagonal and were obtained by a bootstrap procedure with 1000 replicates. NA: values not computed due lack of Fsp sequences.

### CDV subgenotype analysis

In CDV subgenotype analysis, based on criteria for measles *Paramyxovirus* (H amino acid identity of 98% and bootstrap values >70%), we identified at least three subgenotypes in the South America-3 lineage. Subgenotype A included strains 13-CO-12, 19-CO-2012, 26-CO-12, and 1aM-CO-2017 (aa variation 0.2–0.06%); subgenotype B included strains 18-CO-2012, 40-CO-2012, and 44-CO-2012 (aa variation 0.8–1%); and subgenotype C included strains 14-CO-2012 and 39-CO-2012 (aa variation 2.2%) (subgenotypes 3A to 3C in Fig. 2).

Also, we identified three subgenotypes within the “South America/North America-4” lineage. Subgenotype A included strains 16a-CO-2017, 2aP/CO-2017, 13b-CO-2017, and 2a-CO-2017 (aa variation 0.2–1.4%); subgenotype B included strains 13-1941-US-2013 and 13-2262-US-2013 (aa variation 2.6%) (American strains); and subgenotype C included strain 18a-CO-2017 (aa variation 4.3%) (subgenotypes 4A to 4C in Fig. 2).

For evaluating this subclassification for the Fsp-coding region, we arbitrarily extrapolated the classification and found five subgenotypes in the South America-3 lineage (3A to 3E in Fig. 1). Subgenotype A included strains 42-CO-2012, 1aM-CO-2017, and BUC-12-CO-2016 (aa variation 1.2–2.5%) and subgenotype B included strains 18-CO-2012 and 44-CO-12 (aa variation 2–3.5%); both of these subgenotypes were also found with the H gene. Two other subgenotypes were not characterized with the H gene: subgenotype D, including eight Bogotá 2015 strains (aa variation 4.7–5.7%) and subgenotype E, including strains BOG2-CO-2014 and BOG11-CO-2015 (aa variation 5.5–5.7%).

Likewise, in the Fsp analysis of the “South America/North America-4” lineage, we observed four well-defined subgenotypes (4A to 4D in Fig. 1). Subgenotype A included strains 16-CO-2017, 2aP/CO-2017, 13b-CO-2017, 9-CO-2017, and 2-CO-2017 (aa variation 0–1.5%); subgenotype B included strains 13-1941-US-2013, 13-2262-US-2013, and 19-CO-2017 (aa variation 5.5–11.2%); and subgenotype D included Ecuadorian strains not characterized with the H gene (aa variation 1.5–2%).

### Amino acid analysis of the H protein

Analysis of the deduced amino acid sequences of the full-length H protein (607 amino acids) of Colombian CDV viruses showed the presence of exclusive substitutions also found in the South America-3 (N261S, G488R, T544S) and the “South America/North America-4” lineages (E333V, T348K). Remarkably, we found a set of substitutions that are common to both Colombian lineages (T193I, V198I, E333V, S343L, T348K, and A365T); however, the substitutions in the South America-3 lineage appear in the new sequences and not in the previously reported 2012 sequences.

Colombian strains showed the same residue, isoleucine, at position 506 as vaccine strains belonging to the America-1 lineage. Several substitutions in Colombian strains were also present in other wild-type strains reported in different lineages: S22R (America-2), V41I (European Wildlife), N128S (Africa-1), K281R (America-2), G314S (Asia-2 and America-2), and I315V (European Wildlife). All Colombian South America-3 lineage sequences carried asparagine at position 530, a highly variable residue linked to interspecies transmission of the virus (McCarthy *et al*. 2007). The South America-4 sequences included a serine at position 530, while North America-4 displayed an aspartic acid in this position. The H sequences of the 2017 “South America/North America-4” lineage displayed the following unique substitutions: Q5R, L38S, T193I, V198I, V235I, T291M, E333V, H339D, S341L, T348K, and F353I.

In the present study, three sequences from three dogs that had not been completely vaccinated were grouped in the “South America/North America-4” lineage; one strain had a very extensive branch (Fig. 1; sequence Mde-19a-CO-2017), while another was shown to be related to CDV strains circulating in North America (Fig. 2; sequence Mde-18a-CO-2017), and the final strain, 1aM-CO-2017, was shown to belong to the South America-3 lineage (Fig. 2). A comparison of the linear hemagglutinin noose epitope (HNE) between the vaccine strains and these Colombian strains showed the presence of multiple substitutions (Supplemental material Fig. S2). Sequence 18a-CO-2017 presented the following substitutions: A367V, E372D, G376N, and T386S, while strain 1aM-CO-2017 of the South America-3 lineage presented A367V, G376N, and T386S.

### Amino acid analysis of the Fsp peptide

The Fsp peptide possesses 95 variable amino acids (of 135 total). We found 16 substitutions in its sequence that are exclusive to the South America-3 lineage: S9P, T32I, A35S, T40P, D54N, R55K, S58N, Y59H, M61T, R67F H80C, H83R, I102S, Q115H, C116F, and L129F. In the Fsp peptide of the “South America/North America-4” lineage, 20 exclusive substitutions were found: D28S, E29G, A35T, N62D, S71G, H80R, V94I, N108T, S112P, S114P, and K134E. Furthermore, a different set of amino acid substitutions with a specific geographic pattern were found only in South America-4: T13V, T40K, S45F, S58Q, V79I, S95P, R105W, and G113C. In the most identical strains “South America/North America-4” we only found the substitutions T13M and N76S, indicating autapomorphic characteristics.

### CDV H and Fsp glycosylation analysis

Potential glycosylation sites for the CDV H protein from the South America-3 lineage have previously been reported^17^. No new potential glycosylation sites were found in the 2017 samples belonging to this lineage in new sampling areas in Colombia. For the “South America/North America-4” lineage, we identified the presence of eight potential glycosylation sites (NXS/T) at positions 19–21, 149–151, 309–311, 391–393, 422–424, 456–458, 587–589, and 603–605, which are common to other lineages. The previously reported Asia-1 potential glycosylation sites at positions 584–586^41^ were not present in the sequences of the “South America/North America-4” lineage.

Regarding the potential glycosylation analysis of the Fsp peptide of the South America-3 lineage, we found the presence of two potential glycosylation sites (NXS/T) at positions 62–64 and 108–110 that are common to the other lineages. However, no potential glycosylation sites were found in the any of the “South America/North America-4” sequences.

### CDV H and Fsp sites under positive selection

We evaluated non-neutral selection using the FUBAR method. For the H gene, we found that sequences harbored three sites under positive selection: 522, 549, and 582, with a posterior probability of 0.9 and a Bayes factor of 28.3, 220.9, and 44.7 respectively. We also found 247 sites under negative selection with a posterior probability of 0.9 and Bayes factor < 1.

In the Fsp-coding region, we found nine sites under positive selection using the FUBAR method: 21, 39, 46, 51, 79, 98, 99, 101, and 102, with a posterior probability of 0.9 and a Bayes factor of 67, 306, and 88 in sites 21, 76, and 98, respectively. Conversely, we found five sites under negative selection: 45, 83, 89, 121, and 133, with a posterior probability of 0.9 and a Bayes factor < 1.

### H gene and Fsp phylogeography

**Figure 3:**
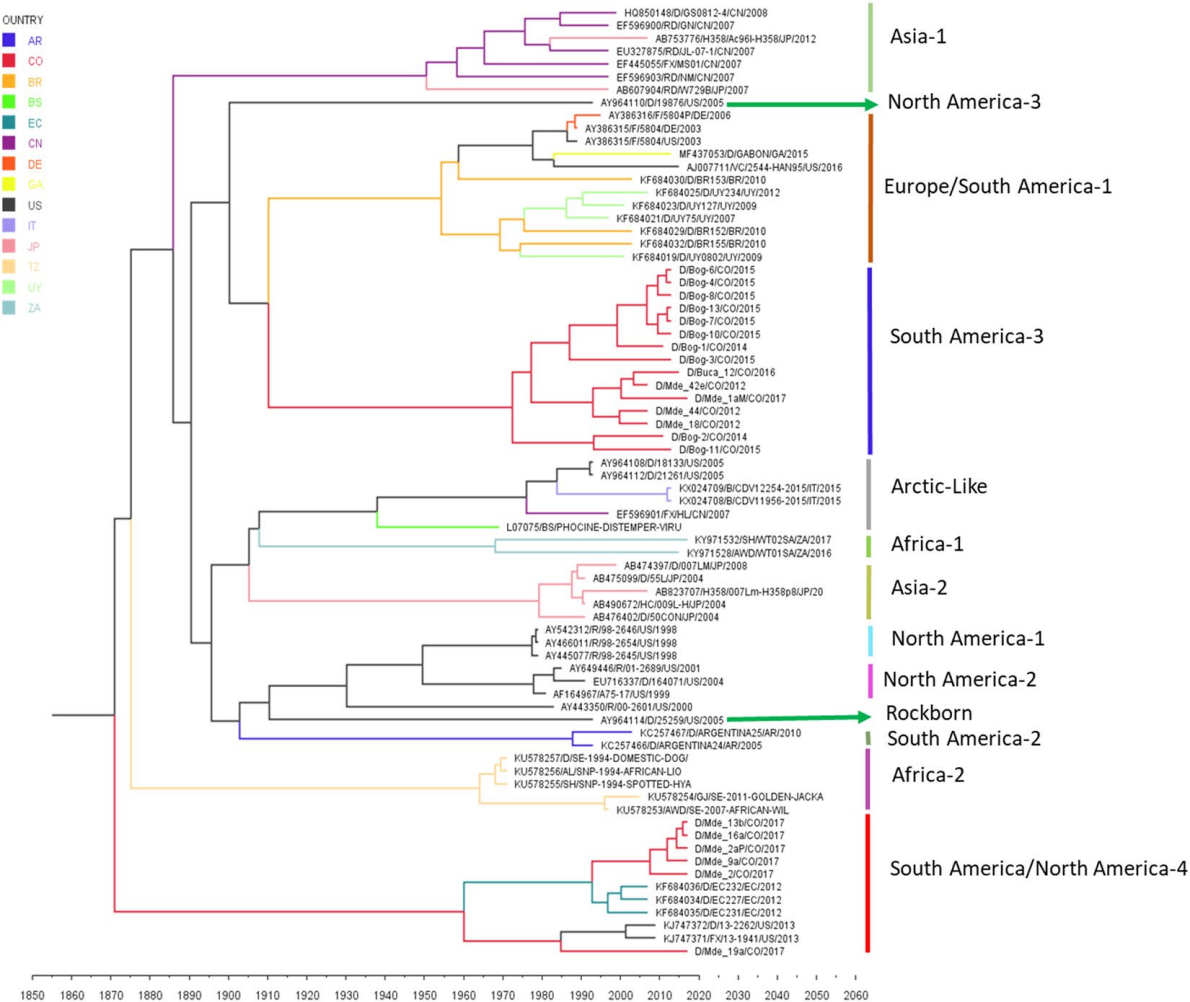
Time-measured Bayesian MCC tree for CDV Fsp fragment. Branches are colored according to the country color code in the upper left. Colombian sequences are depicted in red.

**Figure 4:**
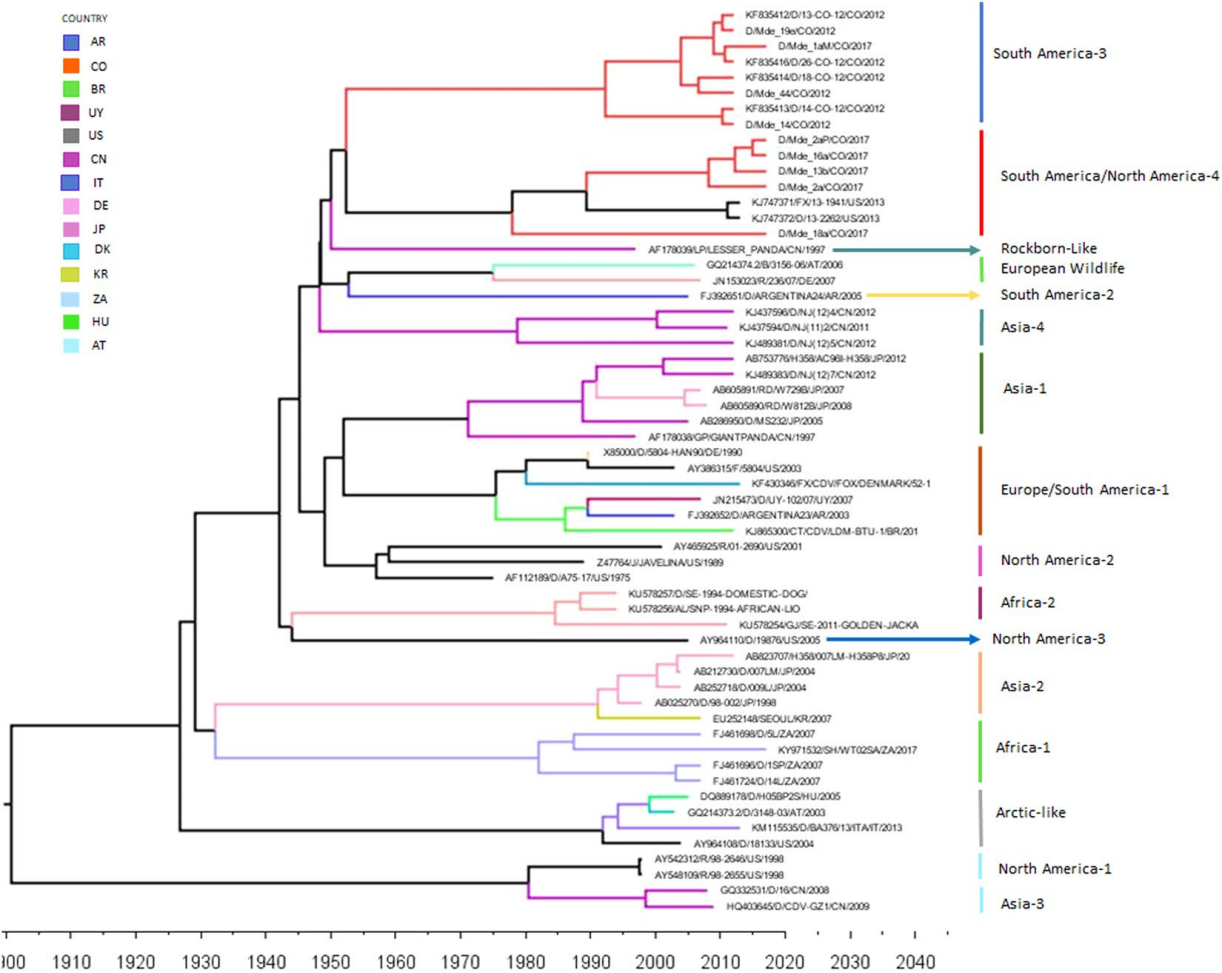
Time-measured Bayesian MCC tree for CDV H gene. Branches are colored according to the country color code in the upper left. Colombian sequences are depicted in red.

For this analysis, the vaccine sequences were eliminated since there were no exact dates of isolation for these strains, which would have produced biases in the substitution rates. Table 4 shows the evolutionary model for each gene. Also, Table 5 shows the estimated TMRCA for the H gene. The South America-3 and “South America/North America-4” lineages have a TMRCA corresponding to 1964 and 1925, respectively (see Table 5). The phylogeography of the Fsp and H gene is shown in Figs 3 and 4, respectively.

**Table 4:** Evolutionary Parameters in Gene H and Fsp coding region.

	Evolution model	Evolution rate		Rate dN/dS	tMRCA	HDP 95% Interval
		Mean	HDP 95% Interval			
H	T92 + G	4,87 × 10^−4^	3,78 × 10^−4^–5,94 × 10^−4^	0,024	1900	1873–1925
Fsp	HKY + G	1,642 × 10^−3^	1,12 × 10^−3^–2,12 × 10^−3^	0,4	1942	1914–1964

**Table 5:** H gene molecular clocks in different CDV lineages.

Lineage	tMRCA		N° Sequences
	Mean	HDP 95% Interval	
North America-1	1998	1996–1997	2
North America-2	1957	1947–1965	3
North America-3	2005	n/a	1
Asia-1	1971	1961–1980	6
Asia-2	1991	1986–1996	5
Asia-3	1999	1994–2003	2
Asia-4	1979	1969–1988	3
Africa-1	1982	1973–1991	4
Africa-2	1985	1979–1990	3
Arctic -Like	1992	1986–1997	4
European wildlife	1975	1965–1985	2
Rockborn-Like	1944	1932–1956	2
Europe/South America-1	1975	1966–1982	6
South America-2	2005	n/a	1
South America-3	1964	1943–1983	8
South/North America-4	1925	1891–1955	7

## Discussion

Phylogenetic characterization of CDV is performed on the basis of the H gene sequence because this gene shows high nucleotide variability in CDV between field strains and vaccine strains in comparison with other paramyxoviruses^42,43^. Using this method, it is accepted that two strains belong to the same lineage when their amino acid diversity is less than 4%^42^. Presently, there are 17 known lineages worldwide with a geographical distribution pattern. However, a fragment that codes for the Fsp of CDV has been suggested as an alternative for the classification of CDV strains since it is highly divergent and has given similar classification results to the H gene. Using this approach, two strains are considered to belong to the same CDV lineage if their amino acid divergence is less than 19%^10^.

In 2012, based on samples only from Medellin city^17^, it was characterized the South America-3 lineage by using the full H gene sequencing approach. However, it was not possible to compare the circulation of this CDV lineage with viruses from other regions of Colombia or in neighboring countries. In the present study, the Fsp-coding region was sequenced from samples obtained in Medellín between 2012 and 2017 and from other Colombian cities such as Bucaramanga (2016) and Bogotá (2015), establishing the wide circulation of the CDV South America-3 lineage in these regions (Fig. 1). In agreement with Sarute *et al*.^10^, the amino acid divergence among the strains (1.5–6.7%) showed that they belonged to the same lineage (data not shown).

Through the phylogenetic analysis of the Fsp-coding region of the present study, we observed that the sequences of the Ecuadorian strains and the former called “America-4” lineage formed a monophyletic group, evidencing a 12% amino acid divergence, which was supported by the table of distances (Table 3). This led us to the conclusion that those viral sequences belong to the same lineage. However, for confirming this finding, we try to amplify the H gene of the same samples. In the resulting phylogenetic tree, we observed a monophyletic group of the strains of the lineage America-4 And also the Ecuadorian strain, which harbored an amino acid divergence in the H gene of 2.8%, showing that these strains belong to the same CDV lineage. For this reason, we suggest calling this lineage “South America/North America-4.”

For comparing the topologies of the phylogenetic trees, we used the North America-1/Vaccine lineage as an outgroup. We observed that although the lineages characterized in this work are sister groups, they present different ancestry. In the H gene tree, the oldest clades are the Africa-1, Arctic-like, and Asia-2 lineages. From this node, the Asia-4, South America-3, and “South America/North America-4” lineages appear as sister groups. A polytomy emerges, from which lineages emerge as sister groups: North America-3 and South America-2; North America-2, European Wildlife, Asia-1, and Rockborn-like; and Africa-2 and Europe/South America-1.

In the Fsp-coding region tree, the ancestral clades of the characterized lineages arise from a polytomy from which sister groups originate: Arctic-like and Asia-2; Africa-1; North America-1 and North America-2; and Rockborn-like (Fig. 1). From this node, another polytomy emerges, giving rise to more sister groups as follows: the Europe/South America-1, South America-2, and Africa-2; and Asia-1, North America-3, South America-3, and “South America/North America-4.” In their 2013 study, Sarute *et al*. described topological differences between the Fsp and H gene trees^10^. Although the topological structures they identified did not entirely correspond with those found in the present study (Figs 1 and 2), it should be taken into account that nowadays, there are a higher number of Fsp coding sequences available, representing most of the lineages characterized to date; besides, Fsp-coding region sequences could now be obtained from Genbank full CDV genomes. However standard phylogenetic studies still been done with the H gene, thus having a greater number of sequences available in the Genbank.

On the basis of the classification system for measles, a subgenotype consists of H gene sequences that have an amino acid identity of 98% and a high bootstrap value (>70%)^28^. On the basis of these criteria, both the South America-3 and “South America/North America-4” lineages present three subgenotypes. When we arbitrarily extrapolated this classification to the Fsp region, a different set of subgenotypes was found (subgenotypes A–E for South America-3 and A–C for “South America/North America-4”), although fewer than for Europe/South America-1, which reportedly contains at least eight subgenotypes (A–H), including CDV H gene sequences from at least seven different countries^28^.

In measles, different subgenotypes are not geographically restricted, although some appear to be mainly endemic in different areas of the world^44^. In the present study, it was not possible to determine the geographic pattern of CDV subgenotypes on the basis of the H gene as has been previously reported for the Europe/South America-1 and South African subgenotypes^6,45^ and for measles^46^. However, with the Fsp fragment, distribution patterns can be observed between regions (Fig. 1); subgenotype 3A circulates only in Medellín, while subgenotype 3D circulates only in Bogotá. Also, in the “South America/North America-4” lineage, subgenotype 4A was only reported in Colombian strains and subgenotype 4D in Ecuadorian strains. A higher number of CDV sequences collected from different areas within those countries would be necessary for better understanding of the circulation history of CDV subgenotypes in the Americas.

A temporary pattern of distribution has been reported for some of the Europe/South America-1 and South African subgenotypes as well as for measles virus genotypes and subgenotypes^28,44,46^. Our results showed a similar temporary pattern of distribution in most of the subgenotypes in both the South America-3 and the “South America/North America-4” lineages (Figs 1 and 2). These results must be carefully evaluated, as although they may show a temporary pattern of CDV distribution and a possible strain displacement pattern, sampling bias could be another possible explanation. Routine international determination of CDV lineages and subgenotypes plus molecular surveillance could be useful for gaining a more accurate epidemiological understanding of temporary CDV distribution.

The uncontrolled commercialization of puppies from South America in the USA could be the route of transmission of the “South America/North America-4” lineage in these two regions of the continent. It is important to highlight that this is the second lineage that is reportedly actively circulating in two different continental regions, the first being Europe/South America-1^20^. It is imperative that wider phylogeographic studies of the “South America/North America-4” lineage are conducted to establish its origin and geographical spread throughout the American continent; it may have originated in Ecuador and spread through Colombia to the USA, or vice versa. Since CDV is a re-emerging infection in the USA, with at least five different lineages in circulation^15^, deeper phylogenetic analysis could help in gaining an understanding of the epidemiology of CDV on this continent.

In the present study, an amino acid divergence close to 11% in the H protein was observed between vaccine strains and the lineages South America-3 and “South America/North America-4” (Table 3). CDV is presently recognized as a single serotype^47^ as there is little evidence of antigenic divergence as a result of genetic divergence. Recently, significant differences were reported in the evaluation of neutralizing titers between “South America/North America-4” lineage strains and an America-1 type vaccine strain^27^. Given those results and the fact that multiple recognized CDV cases have been recorded in vaccinated animals^17,21,28,48^, it is necessary to perform wider, updated antigenic analyses of CDV for understanding the antigenic differences between the multiple worldwide circulating lineages and, potentially, to produce a vaccine update that includes most prevalent antigenic types.

In the positive selection analysis, we observed that the “South America/North America-4” lineage possesses a unique substitution (V79I) in the Fsp fragment at a site that is under positive selection; the South America-3 lineage also possesses a unique substitution (I102S) at a site under positive selection. This was determined using the FUBAR method, which assumes that the selection pressure for each site is constant throughout the phylogeny^38^. In this way, it was also determined that sites 98, 99, 101, and 102 of Fsp are under positive selection. These changes must be studied to understand the role of such substitutions in vaccine failures and interspecies host changes.

Likewise, we found in gene H sites 522, 549, and 582 under positive selection coinciding with site 549, which has been proposed as a key in the species barrier jump^4^, however sites 522 and 582 have not been previously reported related to pathogenicity, vaccine failure or species barrier jump, which should be deeply studied to understand the role of this sites in pathogenesis and inter-species transmission.

The linear HNE located at amino acids 364–392 of the CDV H protein is conserved among the morbilliviruses^49^. This is the region of the H protein that is recognized by antibodies^50,51^. Recently, it has been suggested that substitutions in this epitope may interfere with the ability of the vaccine to provide adequate protection against infection with wild-type viruses^15^. As reported recently in the previously called “North America-4” strains, we observed the presence of multiple substitutions in the HNE of CDV viruses in vaccinated animals (Supplemental Material Fig. S2). However, from the bioinformatic approach used in this study, we can only suggest that these substitutions could be interfering with the capacity of the vaccine to neutralize wild strains. For this reason, it is necessary to perform neutralization studies of vaccines with wild strains. Currently, structural biology studies of Colombian CDV viruses are underway in an effort to understand the role of structural substitutions in the HNE epitope of Colombian CDV viruses and their role in viral neutralization. On this way, it is important to consider thate glycosylation sites found in the Fsp region of the South America-3 lineage (62–64 and 108–110) could be involved in the evasion of the response or could be the result of epistatic interactions in the H gene^52^.

CDV has one of the highest-reported substitution rates in the *Paramyxoviridae* family (10.53–11.65 × 10^−4^ substitutions/base/year)^43^. Our results show that CDV circulating in Colombia exhibits high variability and includes two lineages and multiple subgenotypes (Figs 1 and 2 and Table 3). The temporary and geographical scope of our sampling was not sufficient to accurately explain the variability of CDV in the region. However, considering that the H gene has undergone genetic drift in different geographical regions^26^, we hypothesize that selective and nonselective processes may play important roles in the co-circulation of multiple lineages in an area, as has been reported previously^52^.

Phylogeographic analysis of the Fsp region and the H gene shows an evolutionary rate for the H gene similar to that reported by Fischer *et al*.^53^. However, the resulting two trees differ in their topology in such a way that the ancestry of the lineages of interest is very different (Figs 3 and 4). These differences in topology in comparison to other trees^54^ may be due to the fact that in the present analysis we excluded reported vaccine strains because the vaccine strains has been adapted to cell culture and have different evolutionary rates in comparison with CDV wild strains^52^; Also added three newly reported lineages, including “South America/North America-4.” By comparing both trees, we observed apparently, that the “South America/North America-4”lineage circulated first in Colombia and Ecuador, then in the United States, and again in Colombia (Figs 3 and 4). It is unclear if the variation in spatiotemporal sampling of the “South America/North America-4” lineage is more likely due to bias, as has been reported in other viral models^55^. In addition, since we suspected that most of the ancestral Colombian sequences of both trees are immune escape mutants, deeper analyses must be performed to avoid misleading results regarding the dynamics of the “South America/North America-4” CDV lineage.

Outbreaks of CDV occur in endemic and acute epidemic cycles, leading to transmission among susceptible host populations^54^. In the presence of full or partial vaccination, lifelong immunity could lead to the survival of the remaining coexisting lineages driven by nonselective epidemiological processes^44,56^. Our results, based on unvaccinated and/or incompletely vaccinated populations, support this hypothesis (Table 2).

In contrast with measles, the only natural host of which is humans, broad ranges of host species are susceptible to CDV infection, which results in complications in terms of selection pressure for this virus. It is noteworthy that the “South America/North America-4” lineage characterized in the USA was isolated from domestic dogs and foxes, indicating that this lineage has the ability to jump the species barrier^3,21^. In Colombia, there have been reports of CDV infection in wildlife^57^; however, no phylogenetic analysis has been performed in viruses from those infected animals.

Reported substitutions in circulating CDV protein H in wildlife include E276V, Q392R, R519I, I542F, and Y549H, i.e., sites that show the same substitutions in the South America-3 and South America/NorthAmerica-4 lineages, which indicates the potential of these viruses to jump the species barrier. However, there is no statistical association that demonstrates these hypotheses^58^.

In conclusion, we report the co-circulation of two CDV lineages in Colombia: the South America-3 lineage circulating in Medellín, Bucaramanga, and Bogotá, and the concurrent circulation of a new lineage not previously described in the country that mainly infects dogs in Medellín. The latter lineage is evolutionarily related to strains reported in domestic dogs in Ecuador and in domestic dogs and wildlife in the USA. Given the intercontinental circulation of this lineage, we propose to name it “South America/North America-4.”

## Supplementary information

Supplementary material

## Data Availability

The datasets generated during and/or analysed during the current study are available from the corresponding author upon request.
