# Supplementary material for: Phylogenetic evidence of the intercontinental circulation of a Canine distemper virus lineage in the Americas
Source: Sci Rep. 2019 Oct 31;9:15747. doi: 10.1038/s41598-019-52345-9 (PMC6823503; doi:10.1038/s41598-019-52345-9)
Supplement: Supplementary file 1 — Supplementary material [file 41598_2019_52345_MOESM1_ESM.pdf]

Supplementary material

Phylogenetic evidence of the intercontinental circulation of a Canine distemper virus lineage in the Americas

July Duque-Valencia[a¶], Norma R Forero-Muñoz[b¶], Francisco J Díaz[c], Elisabete Martins[bd], Paola Barato[b], Julián Ruíz-Sáenz[a]*

[a] Grupo de Investigación en Ciencias Animales - GRICA, Facultad de Medicina Veterinaria y Zootecnia, Universidad Cooperativa de Colombia, sede Bucaramanga

[b] Corporación Patología Veterinaria (Corpavet), Bogotá, Colombia

[c] Grupo Inmunovirología, Facultad de Medicina, Universidad de Antioquia, Calle 70 No. 52-21, Medellín, Colombia

[d] Universidade de Lisboa, Lisboa, Portugal

[¶]These authors contributed equally to this work

*Corresponding author: Calle 30A # 33-51. Universidad Cooperativa de Colombia, Bucaramanga, Colombia. Phone/Fax: +57-6356624, *Email address*: julianruizsaenz@gmail.com; julian.ruizs@campusucc.edu.co (J. Ruiz-Saenz)

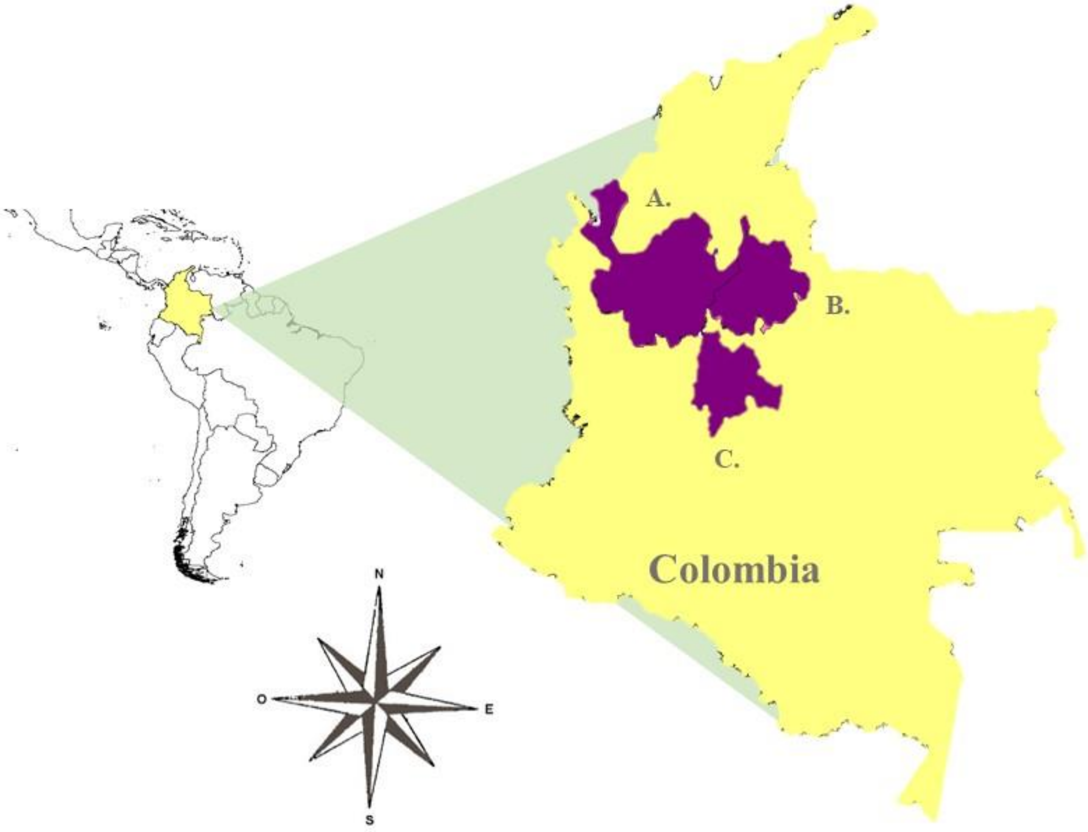

Supplemental Figure 1. Geographical location of sampling sites in Colombia. Purple area denotes the cities of Medellín (A), Bucaramanga (B), and Bogotá (C). The map was created using DIVA-GIS version 7.5.0 for Windows™. See main text for references.

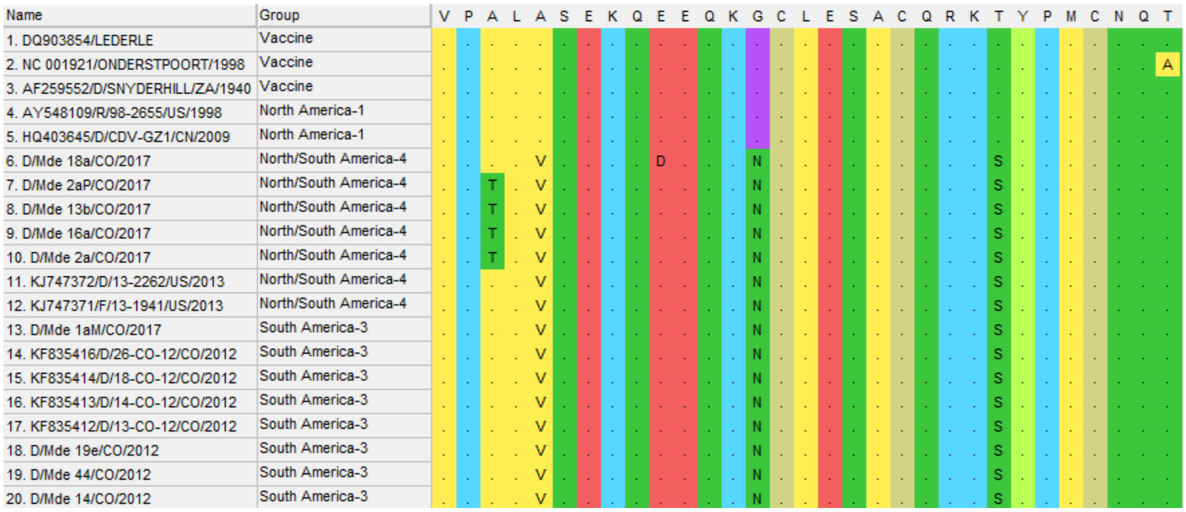

Supplemental Figure 2. Alignment of the hemagglutinating region of the CDV protein H. Alignment of the deduced amino acid sequence (residues 364–392) of the CDV hemagglutinin protein between vaccine strains and the “South America/North America-4” and South America-3 lineages.
